# Patterns and Influencing Factors in the Nurse–Patient Relationship in Hospitals' General Wards: An Integrative Review

**DOI:** 10.1111/scs.70104

**Published:** 2025-09-03

**Authors:** Heleen van Erp, Janneke de Man‐van Ginkel, Anouk Nederend, Anouschka Rense, Meralda Slager, Jet Bussemaker

**Affiliations:** ^1^ Department of Public Health and Primary Care Leiden University Medical Center Leiden the Netherlands; ^2^ Haga Teaching Hospital The Hague the Netherlands; ^3^ Nursing Science, Department of Gerontology and Geriatrics Leiden University Medical Center Leiden the Netherlands; ^4^ Avans University of Applied Sciences Breda the Netherlands; ^5^ Faculty Governance and Global Affairs, Institute of Public Administration Leiden University The Hague the Netherlands

**Keywords:** emotions, hospital, inpatients, literature review, nurse–patient relations, nursing, nursing staff

## Abstract

**Background:**

The nurse–patient relationship is considered important in nursing theories and ethics. Yet, on general hospital wards, such relationships are often not achieved. Prior to addressing developing satisfactory nurse–patient relationships, it is essential to first understand the types of relationships that occur and the dynamics that shape them.

**Aim:**

This systematic integrative review explores the expectations and experiences of patients and nurses regarding their relationship and which mechanisms influence its development.

**Ethics Statement:**

As this study did not involve human subjects, ethical approval was not required.

**Methods:**

A systematic search was conducted in Medline, Embase, PsycInfo and CINAHL (February 2021; targeted update November 2023). Nineteen studies on the nurse–patient relationship in general wards in Western countries were included. Whittemore and Knafl's integrative review methodology guided the process. Data were analysed thematically using Braun and Clarke's approach, and PRISMA guidelines were followed for reporting.

**Results:**

Four central themes were identified: relational needs, force fields, relational abilities and relationship patterns. Relational needs reflect expectations of both patients and nurses. Various force fields can hinder relationship building, while relational abilities influence nurses' interpersonal capacity. Three relationship patterns were found. *Emotionally connected relationships*, marked by mutual emotional involvement, are considered ideal‐typical but difficult to sustain. *Emotionally detached relationships* lack meaningful human connection and often lead to negative experiences. *Socially connected relationships,* characterised by brief but genuine human contact without emotional overexposure, however, meet both nurses' and patients' relational needs.

**Conclusion:**

While emotionally connected relationships are difficult to achieve and emotionally detached ones are undesirable, socially connected relationships offer a feasible and meaningful alternative on hospital wards. Strengthening relational skills, professional identity and valuing human connection within institutional constraints can enhance nursing practise, inform education, guide relational care policy and foster ward cultures that prioritise person‐centred care.

## 1 Introduction

Nursing is widely regarded as a relational profession, with the nurse–patient relationship at its core [1, 2]. This relationship is considered inherently positive and therapeutic [3], and fundamental to the delivery of high‐quality care [4]. Its significance extends beyond individual encounters: international health policy frameworks, including the World Health Organization's agenda for people‐centred and integrated care, emphasise that respectful, participatory relationships between healthcare providers and recipients are critical for achieving equitable, trustworthy and responsive health systems [5]. Strengthening the relational dimension of nursing thus contributes not only to professional ideals but also to tangible patient outcomes, as well as broader societal goals for healthcare delivery.

The centrality of the nurse–patient relationship is firmly embedded in nursing theory, care models and nursing ethics. Many nursing theories regard the relationship as essential for understanding and addressing patients' needs [6], while person‐centred care models emphasise relational engagement as a prerequisite for providing individualised and responsive care [7]. Together, these theories and models underscore the importance of the nurse–patient relationship not only for ensuring high‐quality care but also ground it in ethical principles. Furthermore, point‐of‐care frameworks such as the Fundamentals of Care emphasise that relational engagement is essential for integrating the physical, psychosocial and relational dimensions of care [8]. This framework provides a contextually relevant lens for understanding relational engagement in hospital care [9].

In their concept analysis, Allande‐Cussó et al. [10] characterised a good nurse–patient relationship as a helping relationship, meaning that a union or bond is established. This ideal‐typical nurse–patient relationship is also referred to as a therapeutic relationship. Key attributes of this ideal‐typical relationship include trust, mutual respect, presence, empathy, compassion and shared vulnerability. These relational qualities are not only fundamental to therapeutic engagement but are also essential to the delivery of effective nursing care [11]. Moreover, the desire to engage in therapeutic relationships aligns closely with nurses' intrinsic motivations for entering the profession [12].

Empirical research further supports the link between high‐quality nurse–patient relationships and positive outcomes for both patients and nurses. Reported benefits include reduced vulnerability [13], enhanced mental and physical well‐being [14], improved immunity [15], increased patient involvement and advocacy [16, 17] and higher work engagement and affective commitment to the hospital among nurses [18]. Conversely, low‐quality relationships have been associated with adverse effects, such as moral distress [19].

Despite this evidence, such high‐quality nurse–patient relationships are not consistently realised in hospital settings [20, 21, 22], particularly on general wards [19, 23, 24]. Nurses frequently report organisational constraints on relational engagement, and ‘comfort/talk with patients’ is among the most commonly omitted care activities in European hospitals [25]. These omissions suggest that the relational dimension of nursing is structurally compromised, contributing to a gap between professional ideals and everyday practice [1].

To address this gap, insight is needed into how nurses and patients perceive and experience their relationships within general ward contexts. Specifically, little is known about what both groups consider a satisfactory relationship and what mechanisms shape its development in these settings.

### 1.1 Aim

This review aims to explore patients' and nurses' expectations and experiences regarding the nurse–patient relationship on general hospital wards, as well as the underlying mechanisms that influence its development.

## 2 Methods

### 2.1 Design

A systematic literature review was conducted using an integrative review approach. As this review used publicly available literature, ethical approval was not required. Both empirical and theoretical publications were included to broadly represent the complex concept of the nurse–patient relationship [26]. Borderline inclusion decisions were resolved by team consensus for consistency and fairness in study selection. To enhance scientific rigour, the updated methodology of Whittemore and Knafl [27] was used. This review was reported in accordance with the PRISMA guidelines [28].

### 2.2 Search Methods

We conducted a systematic literature search via Medline (PubMed), Embase (Ovid), PsycInfo (Ovid) and CINAHL (EBSCO). The search strategy was developed in consultation with a medical information specialist. The search included thesaurus terms and free‐text words, including synonyms and closely related words, for the following concepts: ‘caring nurse–patient relationship’, ‘nursing in general hospital wards’ and ‘building and/or establishing a relationship’ (Appendix A: ‘Search strategy’). Electronic databases were searched without any restrictions up to February 19, 2021. Two reference articles were used to validate the search strategy: those by Berg et al. [29] and Suikkala et al. [30].

The initial search resulted in 6641 retrieved database records. After deduplication, 3797 records remained, which were uploaded into the Rayyan QCRI tool [31] for screening.

### 2.3 Inclusion and Exclusion Criteria

The inclusion criteria were developed through discussions among three researchers (HE, AN and AR) (Table 1). Eligibility criteria were formulated with regard to the research setting. Only studies conducted in general wards, defined as inpatient units in general hospitals providing care to adults with physical conditions not requiring intensive or specialised care, were included in the analysis. In these wards, nurse–patient relationships form during short, intermittent and infrequent care encounters [32]. Studies conducted in other types of wards, such as intensive care units, emergency rooms and short‐stay wards, were excluded, as relationships in these settings are likely to develop differently because of the type of care provided or the shorter duration of contact. Similarly, research on relationships in other healthcare settings, such as psychiatry, homecare or nursing homes, was excluded because relationships are shaped by contextual and situational factors [2, 16]. Moreover, only studies involving patients without cognitive impairments, communicative problems or specific relational needs were included. Articles had to specifically report on relationships between nurses and patients.

**TABLE 1:** Inclusion/exclusion criteria.

	Inclusion	Exclusion
Sample	Adult patients	Children/parents
	Registered nurses, bachelor nurses, licensed practical nurses, student‐nurses	Nursing aides, nursing assistants
Setting	General (somatic) hospitals	Other healthcare settings (e.g., psychiatry, homecare, nursing homes, primary care, tertiary care, private hospitals)
	General nursing wards (e.g., internal medicine, cardiology, pulmonology, surgery)	Specialised wards (e.g., emergency, intensive care, obstetrics, paediatrics, daycare, outpatient clinic)
	Research conducted in ‘Western countries’: Northern and Western Europe, USA, Canada, Australia, New Zealand	Research conducted in other countries
Phenomenon of Interest	Relationship between nurse or nursing student and patient	Other relationships (e.g., patient‐physician, patient‐paramedic, patient‐midwife, triadic relationships)
	Patients without specific communication or relational needs, or cognitive impairments	Patients with cognitive impairments (e.g., dementia, delirium, psychiatric disorders, intellectual disability)
		Patients with specific underlying syndromes or special relational needs (e.g., inmates, depressed patients)
		Communication issues (e.g., non‐native speakers)
	Neutral or common nursing situations	Highly emotionally charged or uncommon nursing situations (e.g., end‐of‐life care, terminal care, isolation, unrecognizability of the nurse, for example due to wearing a face mask)
Research type	Empirical articles post‐2000, theoretical articles, position papers	Review articles, dissertations, articles not available as full‐text, articles not written in English or Dutch

Beyond the setting and population, criteria known to influence relationship building were also formulated. Culturally based care beliefs, values and practices can affect nurse–patient relationships [33]. To ensure consistency in socio‐cultural norms and nursing practices, only studies conducted in countries with similar cultural characteristics, referred to as ‘Western countries’, were included. We also limited the review to empirical research conducted from the year 2000 onward, as the average length of hospital stay decreased significantly around that time [34], and there is evidence that relationships develop over time [35].

### 2.4 Search Outcome

All 3797 records were independently screened by title and abstract by two researchers (HE, and either AN or AR) using the Rayyan QCRI tool in ‘Blind‐on’ mode to minimise bias [31]. Discrepancies were resolved through consensus meetings involving these three authors. A total of 110 records were judged eligible for full‐text screening. One paper, known to the authors but not retrieved in the database search for reasons we could not identify, was manually added, resulting in 111 records for full‐text screening. Disagreements (*n* = 18; 16%) and uncertainties (*n* = 34; 31%) were discussed among all three researchers until consensus was reached, resulting in the inclusion of 19 articles.

**FIGURE 1:**
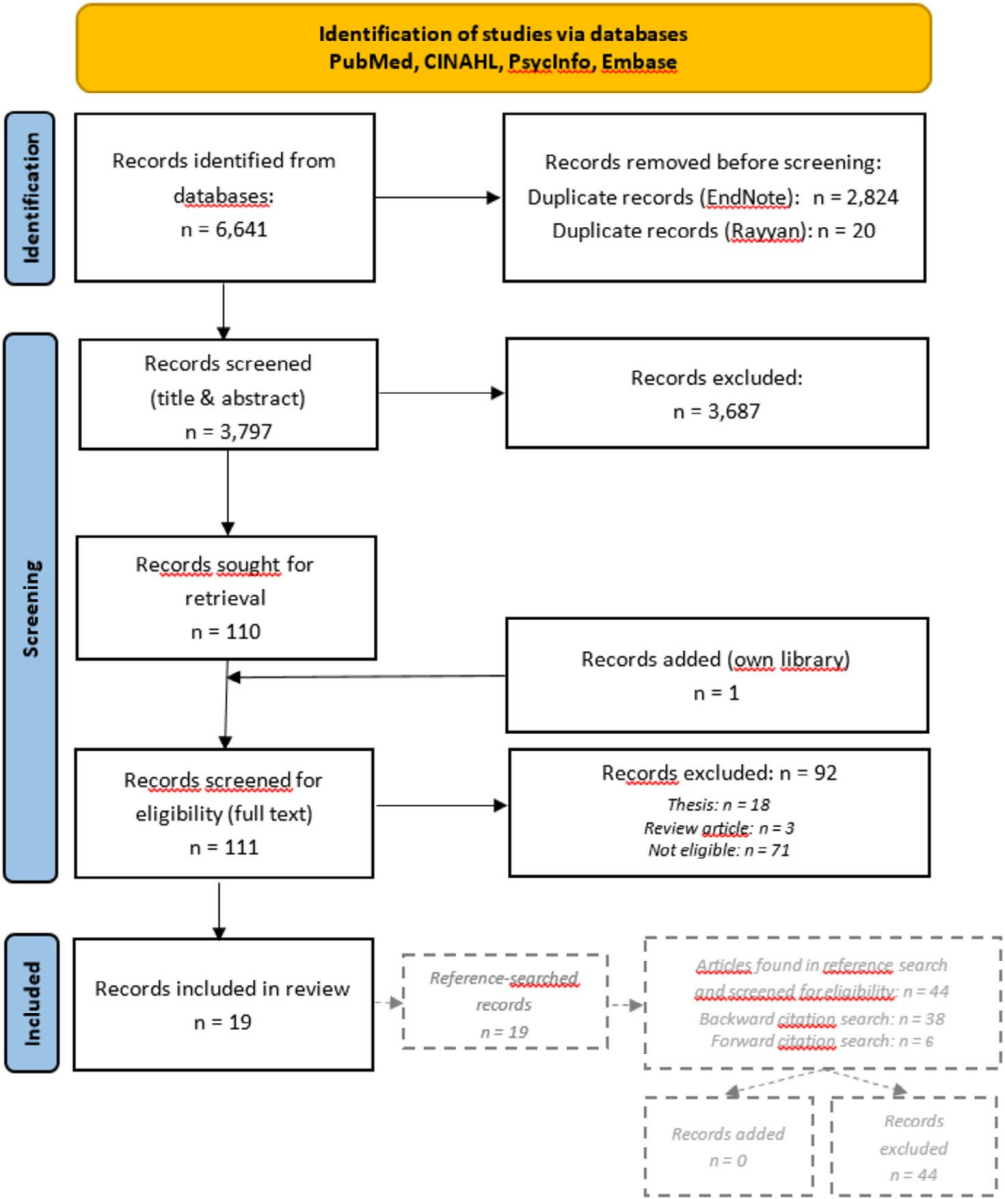
Literature search, PRISMA flow diagram [28].

A backward citation search was then conducted by two researchers independently on these 19 articles (HE, and either AN or AR). Additionally, a forward reference search was conducted on the articles that were rated as highly relevant and of sufficient quality (Appendix B ‘Data evaluation scores’). This yielded 44 records for further consideration, but none met the inclusion criteria. Therefore, the final dataset consisted of 19 articles. The study selection and inclusion procedure is outlined in a PRISMA flow diagram (Figure 1).

The relevance of the included articles was determined independently by two researchers (HE and AN) with a score of ‘high relevance’ or ‘medium relevance’, on the basis of the expected contribution to answering the research question. Agreement between reviewers was high, with only one article having a difference in score (Appendix B ‘Data evaluation scores’).

### 2.5 Methodological Quality Appraisal

Three assessment tools were used to evaluate methodological quality, appropriate for the design of the included articles [36, 37, 38] (Appendix B: ‘Data evaluation scores’). Two researchers (HE, and either AN or AR) independently conducted the primary assessment. The principal investigator (HE) then made an overall judgement, resulting in three methodological quality categorizations: high, medium and low (Appendix B: ‘Data evaluation scores’). The quality and relevance scores were then used to determine which articles would be selected first for initial analysis.

### 2.6 Data Abstraction and Synthesis

The dataset was analysed using the Braun and Clarke [39] thematic analysis method, which involves six recursive phases: familiarisation, coding, generating initial themes, reviewing and developing themes, refining, defining and naming themes and writing.

The eight higher‐quality articles were analysed first. HE, AN and AR individually read the articles to gain an understanding of the content, and then independently coded the meaningful fragments using the qualitative analysis tool ATLAS.ti [40]. Subsequently, the coding and interpretations were discussed in team meetings to facilitate a common understanding of the data. Any differences in interpretation were resolved through discussion until consensus was reached. This process led to the iterative development of a data‐driven codebook. After that, an analysis was conducted to identify themes. To address unintended blind spots and refine the themes, these initial themes were discussed twice with other researchers familiar with healthcare but not working in hospitals. The remaining 11 articles were then analysed. The analysis was deepened through discussions within the research team by exploring relationships between themes in more detail. Throughout this process, all interpretative differences were resolved through team discussion. The final results were reviewed by supervisors as part of the internal quality control process to ensure clarity and completeness in reporting.

Before finalising the manuscript, an updated search in PubMed and CINAHL was conducted (February 2021–October 2023) to check whether new evidence related to nurse–patient relationships had emerged since the original search. These databases were selected because 18 of the 19 initially included articles were retrieved from them. This update yielded two additional studies [41, 42], but these did not provide new insights beyond those already captured in the existing analysis.

## 3 Results

### 3.1 Description of the Included Articles

This review included 19 articles published between 2000 and 2020, including 12 from northwestern Europe, four from North America and three from Oceania. Fourteen of these articles involved qualitative research, and two were theoretical in nature, while the data collection further consisted of one case study, one position paper and one quantitative study. From the experimental research, six studies were conducted among patients, four among (student‐)nurses and six with both nurses and patients (Appendix C: ‘Summary included articles’).

### 3.2 Relationship Development

Four themes were identified regarding expectations and experiences of the nurse–patient relationship and the perceived challenges to building a satisfactory relationship in the hospital context. We defined a satisfactory nurse–patient relationship as one in which a mutually acceptable way of relating is established, and both nurses' and patients' relational needs are meaningfully met; acknowledging that such needs may differ and need not be fulfilled symmetrically.

The first theme, ‘relational needs’, pertains to the intrinsic desires of both patients and nurses regarding the nurse–patient relationship. The second theme is called ‘force field’ and includes factors that nurses and patients in the hospital context are subject to when forming relationships. The third theme, ‘relational abilities’, refers to efforts to meet relational needs and achieve a satisfactory relationship. The fourth theme, ‘relationship patterns’, relates to the three distinguishable relationship patterns that arise within these dynamics.

### 3.3 Theme 1: Relational Needs

Relational needs were found to be prevalent among both patients and nurses.

Patients' relational needs centre on the affirmation of their humanity, which is described as experiencing that the nurse is interested in you as a person [43, 44, 45, 46, 47, 48, 49]. It evokes positive feelings, such as feeling safe, confident, special, calm, comfortable, warm, at ease or acknowledged and reduces feelings of anxiety and vulnerability [29, 43, 45, 47, 48, 50]. Meeting the relational needs of patients is facilitated by nurses' willingness to show a piece of themselves without assuming a professional mask (Berg et al. [29]; [46, 48, 49, 51, 52]). Patients' relational needs can be characterised as longing for human‐to‐human contact with the nurse.

Nurses experience conflicting perspectives on their relationship needs. On the one hand, they want to be emotionally close to patients, which is associated with professional standards [46, 52, 53, 54]. On the other hand, nurses feel the need to protect themselves from strong emotions that are associated with close relationships [46, 48, 55, 56]. If nurses are unable to join these perspectives and, consequently, fail to build a satisfactory relationship with the patient, feelings of guilt or emptiness from not being able to realise the art of nursing may result [52, 53, 55, 56, 57, 58]. Thus, nurses' relational needs can be characterised as balancing between emotional involvement and protection.

### 3.4 Theme 2: Force Fields

Relationship building on general wards is shaped by macro‐, meso‐ and micro‐level factors, which together form a force field of systemic and internal pressures influencing nurses' capacity to establish satisfactory relationships. These forces share the common characteristic of failing to recognise or acknowledge the value of relational work [48, 54, 55, 56, 57, 58] and drive nurses toward emotional distance.

#### 3.4.1 Macro Level

At the macro‐level, we found forces in the domains of policy, education and science.

Policy frameworks emphasise performance targets, accountability for quality, risk management and cost control [53, 54, 55, 56]. These demands promote protocol‐driven care [52, 56], which limits the flexibility needed to build nurse–patient relationships. Additionally, the continuing reduction in hospital length of stay further restricts opportunities for relationship building [46, 49, 55]. These developments occur alongside a growing trend toward consumerism, which can compromise reciprocity in the relationship when the patient acts more as a claimant of a service than a recipient of care [43, 55].

In the educational domain, interpersonal communication is taught as a process of acquiring skills, leading nurses to adopt an instrumental rather than a relational approach to interaction [48, 53, 55, 56].

Finally, within science, the dominance of quantitative paradigms prioritises measurable outcomes, resulting in limited attention to less tangible dimensions of care, such as emotional labour [55, 56].

#### 3.4.2 Meso Level

Force fields were also found at the meso‐level, namely, in departmental culture, the work environment and the management and organisation of care. The findings point to a predominant biomedical perspective in the hospital setting, with a strong focus on physical conditions (Berg et al. [29]; [52, 53, 55]). The work environment is characterised by a high workload and increasing responsibilities, causing nurses to feel overwhelmed by their duties and reducing their ability to build relationships [49, 53, 54, 55, 58]. Additionally, nurses have long been encouraged to avoid (over)engagement, managers tend not to value interpersonal interaction, and nurses receive little help in managing their emotions [48, 55, 56]. Care organised by tasks and time schedules, resulting in limited opportunities for contact, further accentuates the barriers to relationship‐building in nursing practice (Berg et al. [29]; [49, 50, 55, 58]).

#### 3.4.3 Micro Level

At the micro‐level, we identified forces within both nurses and patients that can hinder the establishment of satisfactory relationships.

On the nurses' side, key subthemes include professional identity, professional socialisation, time constraints and strategies for managing emotions. These barriers are reinforced by patients' beliefs, such as the tendency to excuse busy nurses due to high workloads.

##### 3.4.3.1 Professional Identity

Nurses often report valuing holistic care, but when faced with a choice, they prioritise observable, physical or administrative tasks over emotional and psychosocial care, favouring technical responsibilities over human aspects such as being kind and engaging in conversation. They consider distancing an important part of professionalism and underestimate the importance of a satisfactory relationship for patients [46, 48, 51, 52, 53, 55, 56, 57, 58].

##### 3.4.3.2 Professional Socialisation

Emotional distancing is further reinforced by processes related to professional socialisation. There is a collective tendency to avoid discussing difficult emotions, and as a result, nurses are unable to recognise their own feelings or assist younger colleagues with them. Over time, there is a risk of losing patient‐centredness and the capacity for empathy (Berg et al. [29]; [46, 48, 54, 55, 56, 58]).

##### 3.4.3.3 Time

Nurses report perceived time constraints as a major barrier [43, 49, 52, 53, 54, 55, 57, 58, 59], although patients highly value the time given to them, especially when they see that the nurse is busy [43, 47, 48, 54, 57].

##### 3.4.3.4 Strategies for Emotion Management

We found that hospital nurses must cope with difficult emotions. Entering a relationship evokes feelings of anxiety and requires significant mental effort [30, 51, 53, 55, 56]. This implies that nurses face not only patients' suffering, but also their own uncertainty about how to handle patients' problems [46, 55, 56, 57]. Nurses protect themselves from strong emotions through numerous strategies related to consciously or unconsciously creating an unsuitable atmosphere for deeper emotions. They do this by making choices in their communication style and behaviour and by using emotional tactics [44, 46, 48, 54, 55, 56, 58]. Examples include keeping encounters short and communicating superficially, avoiding eye contact, behaving formally, passing on the patient's emotional issues to other professionals, and pointing to integrity and privacy as reasons not to talk to the patient. However, no strategies focused on actively coping with these emotions were found, which might be explained by the invisibility of emotional labour [55, 56, 57, 58].

##### 3.4.3.5 Patient Excusing a Busy Nurse

The above force fields are reinforced by patients' beliefs, labelled as the ‘patient excusing a busy nurse’ phenomenon. Although patients report that they receive little attention and miss casual contact, they believe this is not due to the nurses themselves, who strive to do their best, but rather to the fact that nurses have a high workload. This attribution results in relational needs being ignored [43, 45, 48, 54].

### 3.5 Theme 3: Relational Abilities

In addition to these forces that hinder nurses in relationship building, several personal characteristics of nurses influence developing satisfactory relationships. Nurses who are able to interweave instrumental activities with sociorelational skills are better able to build satisfactory relationships with patients [43, 46, 47, 49, 51, 53, 54, 55, 57, 58, 59]. Professional maturity was also found to be a positive factor in building relationships. This is attributed to a better‐developed clinical grasp and advanced communication and technical skills, fostering the interpersonal aspects of care (Berg et al. [29]; [49, 51, 52, 53]). Conversely, professional maturity may also negatively impact relationship building because experienced nurses have more responsibilities and time constraints [48, 54]. This is corroborated by indications that novice nurses are sometimes better at building nurse–patient relationships than more experienced nurses [48, 54, 56].

Patients value certain personality traits in nurses, such as being genuine, open, positive and having high self‐esteem, and often express a preference for a specific nurse on the basis of interpersonal chemistry or personal attributes [45, 47, 49, 55, 57]. Nurses' shyness and low self‐esteem may complicate establishing a satisfactory relationship [46, 49]. This finding was labelled as ‘nurse agreeableness’.

Patients also contribute significantly to relationship building through ‘vigilance’ and ‘overtures’ strategies. Vigilance refers to keeping a close eye on the nurse, not asking for help outside of formal care moments, or subordinating one's needs to those of other patients [43, 45, 48, 50, 54, 59]. Overtures include behaviours such as trying to be liked and pleasing the nurse, for example, by showing interest, asking personal questions and teasing or giving gifts (Berg et al. [29]; [45, 50, 54]).

### 3.6 Theme 4: Relationship Patterns

Given the dynamics of relational needs and contextual factors, three different relationship patterns can be distinguished in general wards: emotionally connected, emotionally detached and socially connected relationships. The effects found in each of these patterns relate primarily to affect rather than care outcomes.

#### 3.6.1 The Emotionally Connected Relationship

We found evidence of nurses expressing a desire to understand the patient, demonstrating through their attitude that they are present and attentive to the patient's needs, and using dialogic communication combined with active listening [43, 44, 45, 46, 47, 48, 49, 50, 51, 52, 54, 55, 57]. As a result, nurses and patients accept and respect one another (Berg et al. [29]; [46, 47, 49, 50, 51, 52]), and mutual self‐disclosure occurs [44, 45, 55]. In this type of relationship, the patient experiences a sense of acknowledgment, wholeness or at‐homeness, and maintains dignity ([43]; Berg et al. [29]; [45, 46, 47, 55]), while the nurse perceives being a good nurse [49, 53, 55, 56].

#### 3.6.2 The Emotionally Detached Relationship

Our data also showed that patients use words such as cold and efficient for nurses. Patients then experience care as a technical intervention. They feel treated as objects, communication is monologic and patients struggle with the fear of damaging the relationship or even retaliation if they complain [43, 44, 45, 48, 49, 51, 52, 53, 54, 55, 56, 57, 58]. This is related to strong negative effects on patients' feelings, such as feeling powerless, anxious or vulnerable, leading to a sense of disconnectedness and depersonalisation, while the feeling of not knowing each other is described by both patients and nurses [43, 44, 45, 46, 47, 49, 50, 51, 52, 54, 55, 57, 58].

#### 3.6.3 The Socially Connected Relationship

We finally found that patients describe nurses as warm, kind, friendly or light‐hearted when expressing positive experiences [43, 45, 47, 48, 49, 50, 51, 55, 56, 59]. Interactions then often involve casual conversation and lead to a pleasant social climate and mutual sympathy [48, 49, 50, 51, 54, 55, 57]. Nurses demonstrate social behaviours that include using social touch, offering hospitality, doing little extras and showing availability. Their communication style is characterised by the use of colloquial language, shared humour and reassuring words ([43, 44]; Berg et al. [29]; [45, 46, 47, 48, 49, 50, 51]). In response, patients feel welcome and less vulnerable ([43]; Berg et al. [29]; [45, 47, 48, 50, 52, 55]).

## 4 Discussion

The aim of this review was to explore the expectations and experiences of both patients and nurses regarding the nurse–patient relationship in general wards and the underlying mechanisms that influence its development.

### 4.1 Relationship Patterns

Patients' expectations are clear: they want to be acknowledged as human beings. This is achieved through genuine, human‐to‐human contact. Nurses, by contrast, hold more ambivalent expectations. Their desire for closeness often conflicts with a need for emotional self‐protection, while institutional pressures further encourage emotional distance.

Reflecting on the three relationship patterns found within these dynamics of relational needs and force fields, we note that the *emotionally connected relationship* is described in the literature as the ideal typical nurse–patient relationship [10, 14]. However, our review indicates that achieving this relationship pattern within the hospital context is challenging. When complex dynamics lead nurses away from the ideal typical relationship pattern, there is a chance of developing a distant, instrumental *emotionally detached relationship*. This phenomenon is confirmed by Bridges et al. [19]. Van Belle et al. [22] further reported that this is the most prevalent relationship type in hospital settings. Our study offers additional insights into this phenomenon by demonstrating the mechanism by which force fields at the macro‐, meso‐ and micro‐levels contribute to its manifestation, and, consequently, to unsatisfactory nurse–patient relationships.

When emotionally connected relationships are difficult to establish and emotionally detached ones predominate, this raises concerns for the quality of care, given the relational foundations in frameworks such as the Fundamentals of Care [8]. With the identification of a third relationship pattern, the *socially connected relationship*, our review provides a new perspective on nurse–patient relationships in general wards. This patient‐initiated pattern addresses patients' needs to feel acknowledged as human beings while also accommodating nurses' need for emotional boundaries. Our finding that a socially connected relationship could be effective in the hospital context is supported by evidence that minimal social interactions can transform impersonal instrumental exchanges into genuine social interactions that produce feelings of belonging and positive affect [60]. Moreover, single intimate interactions are often sufficient and can take place in a relatively short period of time through a process that does not require the nurse to disclose significant personal information [61]. Thus, the socially connected relationship appears to be a promising model, balancing both patient and nurse needs within the constraints of hospital environments.

### 4.2 Handling the Relational Context

While the socially connected relationship may be a promising model, its feasibility is shaped by individual and institutional factors, which we discuss below.

We found that factors at macro, meso and micro levels constrain human engagement. Hospital nurses operate within a healthcare system that prioritises efficiency, measurable outputs and biomedical interventions. In these environments, opportunities for relational attunement are routinely marginalised, even when nurses themselves value it. This reveals a fundamental tension between the relational ideals embedded in nursing theory and system‐imposed mechanisms that depersonalise care.

Time also plays a prominent role in relational dynamics. Nurses often cite a lack of time or felt time pressure as key reasons for not entering into a relationship. Interestingly, patients seem to internalise this logic, refraining from seeking attention to accommodate the perceived busyness of nurses. This shared and socially accepted ‘busy nurse narrative’ legitimises the deprioritisation of relational care. It mirrors patterns of omitted care described in the literature [25] and reinforces the notion that relational engagement is optional or unrealistic in hospital environments. Yet, time is also seen as a major satisfier by patients, with studies showing that even small gestures of attention can have a positive impact [13, 62, 63]. Our review demonstrates that even brief, genuine human interactions can foster satisfactory, socially connected relationships. These findings challenge the professional assumption that meaningful nurse–patient relationships require substantial time to develop [10].

Another factor affecting relational dynamics is the undervaluation of emotions within the hospital context. While our findings show that the emotional impact of a satisfactory nurse–patient relationship is significant, we also found that emotions and relational work are often underappreciated. Professional socialisation tends to emphasise efficiency and accountability, leaving relational aspects of care secondary. As a result, nurses may associate ‘good nursing’ with procedural accuracy rather than human connection, leading to emotional distancing. This distancing is not only an individual defence mechanism but also a response shaped by institutional norms. Our findings align with prior research, which highlights how emotions are often relegated as weak and vulnerable in clinical practice [1, 64]. When the importance of emotions is not acknowledged, nurses may feel disempowered or demotivated to work on building satisfactory relationships. Our observation that novices are sometimes more successful in establishing satisfactory relationships than more experienced nurses supports this notion, possibly because they are less influenced by ‘feeling rules’ and, thus, are better able to make spontaneous, genuine human contact. The devaluation of emotional engagement helps explain why emotionally connected relationships, though ideal‐typical, are often difficult to establish in practice.

The socially connected relationship thus presents a viable alternative, allowing for meaningful interactions without requiring emotional vulnerability. These interactions can be viewed as expressions of sympathy rather than empathy: warm and respectful, yet emotionally contained, allowing nurses to maintain emotional boundaries while still fostering positive relational outcomes. While this model offers a pertinent solution within the constraints of hospital environments, it also emphasises the importance of relational work, encouraging a shift toward valuing human connection in nursing practice. By addressing the limitations imposed by institutional pressures, the socially connected relationship could become a sustainable and effective approach for enhancing the nurse–patient relationship in general ward settings.

### 4.3 Strengths and Limitations

This review focused on relationship patterns in general wards in Western countries. The dataset included articles that studied the nurse–patient relationship from different methods, such as interviews, observations and theoretical perspectives, among nurses, patients or both. This allowed for a broad understanding of relationship formation. To ensure the findings' clinical applicability, the research team consisted of researchers with nursing backgrounds in science, practice and education.

This review studied the relationships between nurses and patients without specific impediments to their ability to form relationships. Notably, relationships are culturally and contextually contingent; therefore, the results of this study are not generalisable to other care settings or populations.

Some potential risk factors for bias were identified. There may be some underrepresentation of articles on emotionally connected and emotionally detached relationships due to selection decisions, such as excluding research on ‘trust’ or ‘dignity’ or topics such as ‘difficult behaviour’ or ‘aggression’. Nevertheless, we believe that a balanced dataset was constructed because adjacent concepts and negative behavioural elements appeared in the coding. Furthermore, many of the included studies were of moderate or low quality. This has implications for the robustness of the results and points to the need for additional, high‐quality practice‐based research to further substantiate the findings. Finally, grey literature was excluded. As a result, practice‐based experiential knowledge of nurses and patients may have been missed in this review.

### 4.4 Suggestions for Future Research

Building on the relationship types and underlying dynamics identified in this review, future research should examine both how satisfactory nurse–patient relationships can be developed and how different relationship types affect clinical, psychological and experiential outcomes for patients and nurses. Comparative studies across settings and cultures can clarify universal versus context‐specific dynamics. Further investigation is also needed into the force fields that constrain relational behaviours. This could inform targeted strategies in education, leadership and team culture. The role of frontline nurse leaders in supporting relational care warrants closer examination.

Another important direction is exploring how nurses develop relational awareness, both during formal education and in clinical practice. While structured training may risk framing these skills as techniques, informal approaches such as reflection, peer dialogue and storytelling may better support genuine relational engagement. Future studies could assess how current curricula facilitate this and identify opportunities for enhancement.

Finally, future studies should explore how professional ideals and institutional structures shape the value placed on relational work. As the nurse–patient relationship underpins the ethical core of nursing, such insights could guide not only practice development but also broader policy aimed at safeguarding person‐centred care and supporting both staff and patient well‐being.

## 5 Conclusion

This review highlights that social contact and nurses' ability to show a piece of themselves are valuable to patients. This is important for developing a satisfactory relationship that otherwise would not occur or would only occur with considerable effort by both nurses and patients. Building such a relationship, characterised by social, informal, non‐instrumental interaction with human‐to‐human contact, requires relational skills that nurses must develop. In the process, they must learn to address difficult emotions inherent in nursing and balance distance and closeness. Important topics in this regard are strengthening professional identity and acknowledging values in nursing, particularly genuine human contact.

## Author Contributions

H.v.E.: Lead in conceptualization, analysis, investigation, methodology, project administration, resources, validation, visualisation and writing the original draft. A.N., A.R.: support in analysis, investigation, validation and reviewing of draft. J.d.M.‐v.G., M.S., J.B.: supervision, conceptualization support and review of article.

## Ethics Statement

The authors have nothing to report.

## Conflicts of Interest

The authors declare no conflicts of interest.

## Data Availability

The data that support the findings of this study are available from the corresponding author upon reasonable request. Description of the included articles can be found in ‘Appendix C’.

## Appendix A Search Strategy

### Medline (PubMed), 19 February 2021

**Table:**

Search	Query	Results
#7	Search: **#6 AND #4**	1857
#6	Search: **#5 AND #3**	3422
#5	Search: **#1 OR #2**	21,590
#4	Search: (form*[tiab] OR found*[tiab] OR establish*[tiab] OR maintain*[tiab] OR build*[tiab] OR develop*[tiab] OR emerg*[tiab])	10,220,744
#3	Search: (nursing staff, hospital[mh] OR (nurs*[tiab] AND (hospitals[mh:noexp] OR hospitals, general[mh] OR hospital*[tiab] OR ward[tiab] OR wards[tiab] OR ‘clinical setting*’[tiab] OR ‘care setting*’[tiab])) OR ‘nursing care unit*’[tiab] OR ‘nursing unit*’[tiab] OR ‘nursing department*’[tiab])	138,789
#2	Search: ((‘human interaction*’[tiab] OR ‘human relation*’[tiab] OR personal relation*[tiab] OR personal interaction*[tiab] OR interpersonal relation*[tiab] OR interpersonal interaction*[tiab] OR ‘therapeutic relation*’[tiab] OR ‘patient connect*’[tiab] OR ‘personal connect*’[tiab] OR ‘interpersonal connect*’[tiab] OR ‘human connect*’[tiab] OR ‘caring relation*’[tiab] OR ‘care relation*’[tiab] OR ‘relationship based’[tiab] OR ‘caring interaction*’[tiab] OR ‘caring connect*’[tiab] OR ‘human caring’[tiab] OR ‘interpersonal caring’[tiab] OR ‘personal caring’[tiab] OR ‘relational care’[tiab] OR ‘caring encounter*’[tiab] OR ‘personal encounter*’[tiab] OR ‘interpersonal encounter*’[tiab] OR ‘patient encounter*’[tiab] OR ‘human encounter*’[tiab] OR ‘therapeutic encounter*’[tiab]) AND (patients[mh] OR patient*[tiab]))	11,425
#1	Search: ((nurse patient relations[mh] OR ‘nurse patient*’[tiab] OR ‘patient nurse relation*’[tiab]) AND (interaction*[tiab] OR interpersonal[tiab] OR connect*[tiab] OR encounter*[tiab] OR caring[tiab] OR human[tiab] OR therapeutic[tiab] OR relation*[tiab]))	10,951

Abbreviations: mh, MeSH term; mh:noexp, MeSH term not exploded; tiab, title, abstract, author keyword.

*Truncation.

### Embase Classic + Embase 1947 to 2021 February 19

#### Search Strategy

**Table:**

#	Searches	Results
1	(nurse patient relationship/or (‘nurse patient*’ or ‘patient nurse relation*’).ti,ab,kw.) and (interaction* or interpersonal or connect* or encounter* or caring or human or therapeutic or relation*).ti,ab,kw.	10,472
2	(‘human interaction*’ or ‘human relation*’ or personal relation* or personal interaction* or interpersonal relation* or interpersonal interaction* or ‘therapeutic relation*’ or ‘patient connect*’ or ‘personal connect*’ or ‘interpersonal connect*’ or ‘human connect*’ or ‘caring relation*’ or ‘care relation*’ or ‘relationship based’ or ‘caring interaction*’ or ‘caring connect*’ or ‘human caring’ or ‘interpersonal caring’ or ‘personal caring’ or ‘relational care’ or ‘caring encounter*’ or ‘personal encounter*’ or ‘interpersonal encounter*’ or ‘patient encounter*’ or ‘human encounter*’ or ‘therapeutic encounter*’).ti,ab,kw. and (exp patients/or patient*.ti,ab,kw.)	18,101
3	((Nursing staff/or nurs*.ti,ab,kw.) and (hospital/or community hospital/or general hospital/or non‐profit hospital/or private hospital/or public hospital/or teaching hospital/or urban hospital/or (hospital* or ward or wards or ‘clinical setting*’ or ‘care setting*’).ti,ab,kw.)) or (‘nursing care unit*’ or ‘nursing unit*’ or ‘nursing department*’).ti,ab,kw.	166,393
4	(form* or found* or establish* or maintain* or build* or develop* or emerg*).ti,ab,kw.	14,796,936
5	1 or 2	27,798
6	5 and 3	3186
7	6 and 4	1987

Abbreviations: exp. xxx/, exploded thesaurus term; ti,ab,kw, title, abstract, author keywords; xx/, Thesaurus term.

*Truncation.

### APA PsycInfo 1806 to February Week 2 2021

#### Search Strategy

**Table:**

#	Searches	Results
1	(therapeutic processes/or (‘nurse patient’ or ‘patient nurse relation*’).ti,ab,id.) and (interaction* or interpersonal or connect* or encounter* or caring or human or therapeutic or relation*).ti,ab,id.	19,952
2	(‘human interaction*’ or ‘human relation*’ or ‘personal relation*’ or ‘personal interaction*’ or ‘interpersonal relation*’ or ‘interpersonal interaction*’ or ‘therapeutic relation*’ or ‘patient connect*’ or ‘personal connect*’ or ‘interpersonal connect*’ or ‘human connect*’ or ‘caring relation*’ or ‘care relation*’ or ‘relationship based’ or ‘caring interaction*’ or ‘caring connect*’ or ‘human caring’ or ‘interpersonal caring’ or ‘personal caring’ or ‘relational care’ or ‘caring encounter*’ or ‘personal encounter*’ or ‘interpersonal encounter*’ or ‘patient encounter*’ or ‘human encounter*’ or ‘therapeutic encounter*’).ti,ab,id. and (exp patients/or patient*.ti,ab,id.)	9703
3	((nurses/or nurs*.ti,ab,id.) and (hospitals/or (hospital* or ward or wards or clinical setting* or care setting*).ti,ab,id.)) or (nursing care unit* or nursing unit* or nursing department*).ti,ab,id.	26,881
4	(form* or found* or establish* or maintain* or build* or develop* or emerg*).ti,ab,id.	2,359,891
5	1 or 2	28,277
6	5 and 3	1173
7	6 and 4	721

Abbreviations: exp. xxx/, exploded Thesaurus term; ti,ab,id, title, abstract, author keywords; xx/, Thesaurus term.

*Truncation.

### CINAHL Friday, February 19, 2021 5:07:58 AM

**Table:**

S1	((MH ‘Nurse–Patient Relations’ OR TI (‘Nurse patient*’ OR ‘patient nurse relation*’) OR AB (‘nurse patient*’ OR ‘patient nurse relation*’)) AND (TI (interaction* OR interpersonal or connect* OR encounter* OR caring OR human OR therapeutic or relation*) OR AB (interaction* OR interpersonal OR connect* OR encounter* OR caring OR human OR therapeutic OR relation*)) OR (TI (‘human interaction*’ OR ‘human relation*’ OR ‘personal relation*’ OR ‘personal interaction*’ OR ‘interpersonal relation*’ OR ‘interpersonal interaction*’ OR ‘therapeutic relation*’ OR ‘patient connect*’ OR ‘personal connect*’ OR ‘interpersonal connect*’ OR ‘human connect*’ OR ‘caring relation*’ OR ‘care relation*’ OR ‘relationship based’ OR ‘caring interaction*’ OR ‘caring connect*’ OR ‘human caring’ OR ‘interpersonal caring’ OR ‘personal caring’ OR ‘relational care’ OR ‘caring encounter*’ OR ‘personal encounter*’ OR ‘interpersonal encounter*’ OR ‘patient encounter*’ OR ‘human encounter*’ OR ‘therapeutic encounter*’) OR AB (‘human interaction*’ OR ‘human relation*’ OR ‘personal relation*’ OR ‘personal interaction*’ OR ‘interpersonal relation*’ OR ‘interpersonal interaction*’ OR ‘therapeutic relation*’ OR ‘patient connect*’ OR ‘personal connect*’ OR ‘interpersonal connect*’ OR ‘human connect*’ OR ‘caring relation*’ OR ‘care relation*’ OR ‘relationship based’ OR ‘caring interaction*’ OR ‘caring connect*’ OR ‘human caring’ OR ‘interpersonal caring’ OR ‘personal caring’ OR ‘relational care’ OR ‘caring encounter*’ OR ‘personal encounter*’ OR ‘interpersonal encounter*’ OR ‘patient encounter*’ OR ‘human encounter*’ OR ‘therapeutic encounter*’)) AND (MH ‘Patients+’ or TI patient* OR AB patient*)) AND (MH ‘Nursing Staff, Hospital’ OR ((TI nurs* OR AB nurs*) AND (MH ‘Hospitals’ OR MH ‘Hospitals, Community’ OR MH ‘Hospitals, Private’ OR MH ‘Hospitals, Public’ OR MH ‘Hospitals, Urban’ OR MH ‘Magnet Hospitals’ OR TI (hospital* OR ward OR wards OR ‘clinical setting*’ OR ‘care setting*’) OR AB (hospital* OR ward OR wards OR ‘clinical setting*’ OR ‘care setting*’))) OR TI (‘nursing care unit*’ OR ‘nursing unit*’ OR ‘nursing department*’) OR AB (‘nursing care unit*’ OR ‘nursing unit*’ OR ‘nursing department*’)) AND (TI (form* OR found* OR establish* OR maintain* OR build* OR develop* OR emerg*) OR AB (form* OR found* OR establish* OR maintain* OR build* OR develop* OR emerg*))	2076

Abbreviations: AB, abstract; MH, thesaurus term; MH xx + , exploded thesaurus term; TI, title.

*Truncation.

### Retrieved Articles

**Table:**

	Retrieved articles
Medline (Pubmed)	1857
Embase.ovid	1987
PsycInfo.ovid	721
CINAHL (EBSCO)	2076
	6641

## Appendix B Data Evaluation Scores

**Table:**

	Author/Year	Title	Reviewer	Methodological quality														Relevance scores	
				1	2	3	4	5	6	7	8	9	10	11	12	Quality appraisal	Assessed with:	Relevance score HE	Relevance score AN
1	Andersson et al. (2011)	Experiences of caretime during hospitalisation in a medical ward: Older patients' perspective	HE	Cannot tell	Yes	Yes	Cannot tell	Yes	No	Yes	Cannot tell	No	Yes			Low	CASP‐Qualitative‐Checklist‐2018	Medium	Medium
			AN	Cannot tell	Yes	Yes	Cannot tell	Yes	No	Yes	Yes	Yes	Yes						
2	Barrere (2007)	Discourse analysis of nurse–patient communication in a hospital setting: implications for staff development	HE	Yes	Yes	Yes	Yes	Yes	Cannot tell	Yes	Cannot tell	Yes	Cannot tell			Low	CASP‐Qualitative‐Checklist‐2018	Medium	Medium
			AN	—	Yes	Yes	Cannot tell	Cannot tell	No	No	Cannot tell	Yes	Cannot tell						
3	Berg and Danielson (2007)	Patients' and nurses' experiences of the caring relationship in hospital: an aware striving for trust	HE	Yes	Yes	Yes	Cannot tell	Yes	No	No	Yes	Yes	Yes			Low	CASP‐Qualitative‐Checklist‐2018	High	High
			AN	Yes	Yes	Yes	Cannot tell	Yes	No	No	Yes	Yes	Cannot tell						
4	Berg et al. (2007)	Caring relationship in a context: fieldwork in a medical ward	HE	Yes	Yes	Yes	Yes	Yes	Yes	Yes	Yes	Yes	Yes			High	CASP‐Qualitative‐Checklist‐2018	High	High
			AN	—	Yes	Yes	—	Yes	Cannot tell	Yes	Yes	Yes	Cannot tell						
5	Blockley and Alterio (2008)	Patients' experiences of interpersonal relationships during first time acute hospitalisation	HE	Yes	Yes	Cannot tell	Cannot tell	Yes	No	Yes	Yes	Yes	Cannot tell			Medium	CASP‐Qualitative‐Checklist‐2018	Medium/high	High
			AN	Cannot tell	Yes	Yes	No	Yes	No	Yes	—	Yes	Yes						
6	Caramanzana (2020)	Millennial Nurses Connecting With Patients	HE	Yes	Yes	Cannot tell	Cannot tell	No	No	Cannot tell	No	Cannot tell	No			Low	CASP‐Qualitative‐Checklist‐2018	Medium	Medium
			AN	Yes	Yes	Cannot tell	Cannot tell	Cannot tell	Cannot tell	No	Cannot tell	Yes	No						
7	Cortis (2000)	Caring as experienced by minority ethnic patients	HE	Yes	Yes	Yes	Cannot tell	Yes	No	Cannot tell	No	Yes	Yes			Medium/low	CASP‐Qualitative‐Checklist‐2018	Medium	Medium
			AR	Yes	Yes	Yes	Yes	Yes	No	No	Yes	Yes	Yes						
8	Crary (2016)	Relatedness Matters	HE	Yes	Cannot tell	Yes	Yes	Yes	No							Medium	JBI_Checklist for text and opinion	High	High
			AN	Yes	Yes	Yes	Yes	Yes	No										
9	Crawford et al. (2017)	Tracing the discursive development of rapport in intercultural nurse–patient interactions	HE	Yes	Yes	Yes	No	Yes	Yes	Yes	Cannot tell	Yes	No			Low	CASP‐Qualitative‐Checklist‐2018	High	High
			AN	Cannot tell	Yes	Yes	Cannot tell	Cannot tell	Cannot tell	Yes	Cannot tell	Yes	Cannot tell						
10	Jangland et al. (2011)	Surgical nurses' different understandings of their interactions with patients: a phenomenographic study	HE	Yes	Yes	Yes	Yes	Yes	No	Yes	Yes	Yes	Yes			High	CASP‐Qualitative‐Checklist‐2018	Medium	Medium
			AR	Yes	Yes	Yes	Yes	Yes	Yes	Yes	Yes	Yes	Yes						
11	Kelly et al. (2020)	Patients' experiences of nurses' heartfelt hospitality as caring: A qualitative approach	HE	Yes	Yes	Yes	No	Yes	Yes	Yes	Cannot tell	Cannot tell	Yes			Medium/high	CASP‐Qualitative‐Checklist‐2018	Medium	Medium
			AR	Yes	Yes	Yes	Yes	Yes	Yes	No	Yes	Yes	Yes						
12	Lotzkar and Bottorff (2001)	An observational study of the development of a nurse–patient relationship	HE	Yes	Yes	Yes	No	Yes	Cannot tell	Yes	Cannot tell	Yes	No			Medium	CASP‐Qualitative‐Checklist‐2018	High	High
			AR	Yes	Yes	Yes	Cannot tell	Yes	No	Yes	Yes	Yes	Yes						
13	McCabe (2004)	Nurse–patient communication: an exploration of patients' experiences	HE	Yes	Yes	Yes	Yes	Yes	No	Yes	Yes	Yes	Yes			High	CASP‐Qualitative‐Checklist‐2018	Medium/high	High
			AR	Yes	Yes	Yes	Yes	Yes	No	—	Yes	Yes	Yes						
14	McQueen (2000)	Nurse–patient relationships and partnership in hospital care	HE	Yes	Cannot tell	Yes	Yes	Yes	Yes							High	JBI_Checklist for text and opinion	High	High
			AN	Yes	Cannot tell	Yes	Yes	Yes	Yes										
15	Ramvi (2011)	The risk of entering relationships: experiences from a Norwegian hospital	HE	Yes	Cannot tell	Yes	Yes	Yes	Yes							High	JBI_Checklist for text and opinion	Medium	Medium
			AN	Yes	No	Yes	Yes	Yes	Yes										
16	Suikkala and Leino‐Kilpi (2005)	Nursing student‐patient relationship: Experiences of students and patients	HE	Yes	Cannot tell	No	Yes	Yes	Yes	Yes	Yes	Yes	Yes			High/medium	CASP‐Qualitative‐Checklist‐2018	Medium	Medium
			AR	Yes	Cannot tell	No	Yes	Yes	Yes	Yes	Yes	Yes	Yes						
17	Suikkala et al. (2009)	Factors related to the nursing student‐patient relationship: The patients perspective	HE	Yes	Yes	Yes	Yes	No	Yes	Yes	Cannot tell	Yes	No	Cannot tell	Yes	Medium	Critical Appraisal of a Survey (CEBMa)	High	High
			AN	Yes	Yes	Yes	Yes	No	Yes	Yes	No	Yes	Yes	No	Yes				
18	Uhrenfeldt et al. (2018)	The centrality of the nurse–patient relationship: A Scandinavian perspective	HE	Yes	Yes	Yes	Yes	Yes	Yes							High	JBI_Checklist for text and opinion	High	High
			AN	Yes	Yes	Yes	Yes	Yes	Yes										
19	Vinckx et al. (2018)	Understanding the complexity of working under time pressure in oncology nursing: A grounded theory study	HE	Yes	Yes	No	Yes	Yes	No	Yes	Cannot tell	Yes	Yes			Medium	CASP‐Qualitative‐Checklist‐2018	Medium	Medium
			AR	Yes	Cannot tell	Cannot tell	Yes	Yes	Yes	Yes	Yes	Yes	Yes						

*Note:* green = meets criterion; orange = does not meet criterion; yellow = inconclusive ; gray = no more criteria to answer in this specific instrument.

## Appendix C Summary of Included Studies

**Table:**

Author, Year, Country, Title	Study objective	Participants/Setting	Perspective	Study design, data collection and data analysis
Andersson et al. (2011) Sweden *Experiences of caretime during hospitalisation in a medical ward: older patients' perspective*	To describe older patients' experiences of caretime during hospitalisation in a medica ward	Nine older people, some isolated Hospital ward	Patient	Qualitative research Semi‐structured interviews Thematic content analysis
Barrere (2007) US *Discourse Analysis of Nurse–Patient Communication in a Hospital Setting: Implications for Staff Development*	To examine symmetry (active listening)/asymmetry (dominance) of nurse–patient communication	20 Gendered n/p pairs Two community hospitals	Patient/nurse	Qualitative research, ethnographic study Discourse analysis of taped conversations
Berg and Danielson (2007) Sweden *Patients' and nurses' experiences of the caring relationship in hospital: an aware striving for trust*	To illuminate experiences of the care relationship of long‐term ill patients and their nurses	7 Chronical ill patients, 6 nurses Hospital	Patient/nurse	Qualitative research, empirical study 13 Interviews Interpretive phenomenological analysis method
Berg et al. (2007) Sweden *Caring relationship in a context: Fieldwork in a medical ward*	Examine how the care relationship is established in a medical context	51 Patients, 10 nurses	Patient/nurse	Qualitative research Participant observation with field notes Interpretive phenomenological analysis method
Blockley and Alterio (2008) New Zealand *Patients' experiences of interpersonal relationships during first time acute hospitalisation*	To examine the role of interpersonal relationships on patients' experiences during first time acute hospitalisation	12 Patients	Patient	Qualitative research Semi‐structured interviews and personal stories
Caramanzana (2020) US *Millennial Nurses Connecting With Patients*	To explore and identify what connecting with patients means to millennial nurses	12 Millennial nurses Hospital setting	Nurse	Qualitative research, phenomenological study Semi‐structured interviews, field notes
Cortis (2000) UK *Caring as experienced by minority ethnic patients*	To explore the concept of care, and recent experiences of ‘care’ as delivered by nurses in hospitals with a sample of Pakistani immigrants in Bradford, UK	20 Male and 18 female respondents from Pakistani communities	Patient	Qualitative research Face‐to‐face in‐depth semi‐structured interviews
Crary (2016) US *Relatedness Matters*	To introduce a theoretical approach that exemplifies the importance of relatedness in maintaining the nurse–patient relationship	—	—	Theoretical paper
Crawford et al. (2017) Australia *Tracing the discursive development of rapport in intercultural nurse–patient interactions*	To examine the development of rapport by registered nurses from a variety of cultural and linguistic backgrounds in an Australian hospital	CALD RNs and their patients Day surgery unit and two surgical wards	Nurse/patient	Qualitative research Participant observations and audio‐recordings of interactions Interactional sociolinguistic (IS) and theme oriented discourse analysis
Jangland et al. (2011) Sweden *Surgical nurses' different understandings of their interactions with patients: a phenomenographic study*	To identify and describe how surgical nurses understand and perceive their role in the nurse–patient relationship	17 RN's Two hospitals, surgical units	Nurse	Qualitative research, phenomenographic study Interviews Phenomenographic analysis method
Kelly et al. (2020) New Zealand *Patients' experiences of nurses' heartfelt hospitality as caring: A qualitative approach*	To explore the nature, meaning and experience of hospitality as care from the perspective of elective surgery patients	7 Patients, different cultural backgrounds Private and public hospitals	Patient	Qualitative research, hermeneutic phenomenological methodology Semi‐structured interviews
Lotzkar and Bottorff (2001) Canada *An Observational Study of the Development of a Nurse–Patient Relationship*	To identify features of nurse–patient interactions in the development of a nurse–patient relationship	One patient and one nurse (one dyad) Cancer treatment unit	Nurse/patient	Exploratory descriptive case study Videotaped observations (60 interactions over a 3‐day period) Micro analysis of interactions, using qualitative ethological methods
McCabe (2004) Ireland *Nurse–patient communication: an exploration of patients' experiences*	To research and develop explanations of patients' experiences of the way nurses communicate	8 Patients General teaching hospital	Patient	Qualitative research, hermeneutic phenomenological approach Unstructured interviews
McQueen (2000) UK *Nurse–patient relationships and partnership in hospital care*	Describe the context of nursing care, partnership, forming a therapeutic relationship and acknowledgement of hidden work in forming and maintaining therapeutic relationships	—	—	Theoretical article
Ramvi (2011) Norway *The risk of entering relationships: experiences from a Norwegian hospital*	To examine whether the concept of the social defence system used by Menzies Lyth can still apply to nurses working and being trained in hospitals in Norway	6 Students, 8 nurses, 2 teachers Two medical units at a hospital	Nursing student/nurse/teacher	Qualitative research, fieldwork study Observation of students and their teachers and contact nurses during meetings
Suikkala and Leino‐Kilpi (2005) Finland *Nursing student–patient relationship: Experiences of students and patients*	To explore nursing students' and patients' experiences of their relationship	30 Nursing students, 30 patients Medical ward	Nursing student/patient	Qualitative research Semi‐structured interviews Inductive content analysis
Suikkala et al. (2009) Finland *Factors related to the nursing student‐patient relationship: the patients' perspective*	To describe patients' perceptions of factors related with three types of relationship and to identify which factors predict the type of relationship	277 Patients 10 Hospitals, internal medicine ward	Patient	Quantitative research Five‐point Likert‐type questionnaire Statistical analysis
Uhrenfeldt et al. (2018) Danmark/Norway *The centrality of the nurse–patient relationship: A Scandinavian perspective*	To address ontological and epistemological aspects of importance in the nurse–patient relationship	—	—	Discursive position study
Vinckx et al. (2018) Belgium *Understanding the complexity of working under time pressure in oncology nursing: A grounded theory study*	To study oncology nurses' experiences with time pressure, its perceived impact on nursing care and the ways in which they cope with it	14 Nurses Academic hospital, 5 oncology wards	Nurse	Qualitative research, grounded theory study Semi‐structured in‐depth interviews Qualitative Analysis
